# Development and validation of the caregiver-report version of the international depression questionnaire (IDQ-CG) and international anxiety questionnaire (IAQ-CG)

**DOI:** 10.1007/s00787-024-02495-7

**Published:** 2024-06-18

**Authors:** Enya Redican, Cedric Sachser, Lucy Berliner, Elisa Pfeiffer, Dmytro Martsenkovskyi, Philip Hyland, Menachem Ben-Ezra, Mark Shevlin

**Affiliations:** 1 School of Psychology, Ulster University, Cromore Road, Coleraine, Northern Ireland BT52 1SA UK; 2 Clinic for Child and Adolescent Psychiatry/Psychotherapy, University of Ulm, Ulm, Germany; 3 German Center for Mental Health (DZPG), partner site Ulm, Berlin, Germany; 4 Harborview Center for Sexual Assault and Traumatic Stress, University of Washington, Seattle, WA USA; 5 Department of Psychiatry and Narcology, Bogomolets National Medical University, Kyiv, Ukraine; 6 SI “Institute of Psychiatry, Forensic Psychiatric Examination and Drug Monitoring of Ministry of Health of Ukraine”, Kyiv, Ukraine; 7 Department of Psychology, Maynooth University, Kildare, Ireland; 8 School of Social Work, Ariel University, Ariel, Israel

**Keywords:** Depression, Anxiety, Measurement, Ukraine, ICD-11

## Abstract

The International Depression Questionnaire (IDQ) and International Anxiety Questionnaire (IAQ) are self-report measures of ICD-11 single episode depressive disorder (DD) and generalised anxiety disorder (GAD). The present study sought to describe the development and psychometric evaluation of the caregiver-report versions of the IDQ and IAQ for children, referred to as the IDQ-CG and IAQ-CG, respectively. Participants were 639 parents living in Ukraine who provided data on themselves and one child in their household as part of “*The Mental Health of Parents and Children in Ukraine Study: 2023 Follow-up”* study. The latent structure of the IDQ-CG and IAQ-CG were tested using confirmatory factor analysis (CFA), composite reliability (CR) estimates were estimated, and convergent validity was assessed. Prevalence rates of probable ICD-11 DD and GAD were also estimated. CFA results indicated that the IDQ-CG and IAQ-CG were unidimensional, while the internal reliability of both scales was excellent. Convergent validity was established via associations with external measures of internalizing, externalizing, and attention problems as well as trauma exposure. Factors associated with increased IDQ-CG and IAQ-CG scores included pharmacological support for emotional or behavioural problems, delayed milestone development, being forced to move to another part of Ukraine, serious life disruption due to the war, and having experienced a bereavement. Of the total sample, 1.6% met diagnostic requirements for ICD-11 DD and 5.8% met diagnostic requirements for ICD-11 GAD. This study supports the psychometric properties of the IDQ-CG and IAQ-CG. These measures can be effectively used to identify young people in need of mental health support.

Caregivers possess the most in-depth knowledge of their child’s development and can report on their child in a manner that is sensitive to contextual, historical, and developmental factors [1]. Thus, in instances when children or adolescents are unable to self-report, or when information from a parent is prioritized, a validated caregiver-reported measure is necessary. Examples of situations where parental report is beneficial includes when a child’s reading abilities are inadequate, or when clinicians require additional information to supplement their evaluations of child and adolescent self-report information. One such situation where a caregiver report is necessary is the war in Ukraine. The children and adolescents of Ukraine have endured unimaginable suffering and devastation since Russian forces invaded their country on February 24, 2022. High levels of exposure to death, injury, and displacement, together with disruptions to their education, lack of access to basic resources, and being separated from their families, places millions of Ukrainian young people at risk for mental health problems both now and in the future [2]. To ensure adequate provision of mental health resources for these young people, it is crucial that validated Ukrainian-translated screening instruments are developed [3].

Anxiety and depression are among the most common mental health problems experienced by war-affected young people [5]. One of the few studies to explore the prevalence of depression and anxiety in Ukrainian young people was based on data gathered in the aftermath of the 2014 Russian invasion of eastern Ukraine [4]. Using the Generalised Anxiety Disorder-7 (GAD-7) [6] for anxiety and the Patient Health Questionnaire-9 (PHQ-9) [7] for depression, a recent study [5] found that 23.2–28.9% of Ukrainian young people had mild to moderate anxiety, 1.5–4.4% had severe anxiety, 25.2–28.2% had mild to moderate depression, and 2.9–7.5% had moderately severe to severe depression. Notably, this study utilised the GAD-7 and PHQ-9 which no longer align with current diagnostic descriptions of anxiety and depression, respectively and which do not include child-friendly language.

In the 11th version of the International Classification of Diseases (ICD-11; World Health Organization (WHO) [8] revised formulations of single episode depressive disorder (DD: CODE 6A70) and generalized anxiety disorder (GAD: code 6B00) are included. ICD-11 DD is characterized by a period of low mood or decreased interest in activities, along with other symptoms such as difficulty concentrating, worthlessness, or excessive or inappropriate guilt that occurs most of the day, nearly every day, for a minimum of two weeks and is linked to functional impairment. ICD-11 GAD is characterized by general apprehension or excessive worry surrounding daily life events (such as family, health, finances, school, or work), along with other symptoms such as muscular tension or motor restlessness, concentration difficulties, sleep disturbance that occurs most days, for at least several months, and is associated with functional impairment. Although these diagnostic descriptions also apply to children and adolescents, the ICD-11 recognizes that there may be variations in how symptoms manifest [8]. According to the ICD-11, GAD in young people may manifest as people-pleasing, perfectionism, excessive irritability, somatic symptoms, and excessive preoccupation with rule compliance.

Recently, the International Depression Questionnaire (IDQ) and the International Anxiety Questionnaire (IAQ) were developed as self-report measures of ICD-11 DD and GAD, respectively [9]. The IDQ consists of nine items and the IAQ consists of eight items; both measures include two items which measure ‘essential’ symptoms of both disorders, and the remaining items measure the ‘accompanying’ symptoms. Both measures can be used to identify probable diagnostic cases or to capture symptom severity [9]. Prior studies have provided support for the validity and reliability of the IDQ and IAQ in the general adult population [9], as well as in representative samples of bereaved adults [e.g., 10;11]. Specifically, the structural validity of the IAQ and IDQ has been supported through the identification of a unidimensional model as the best-fitting model [9, 10], and convergent validity was supported through associations with external variables [10, 11]. Notably, no developmentally appropriate versions of the IAQ and IDQ which parents/caregivers can complete on behalf of their child have been developed. The availability of such measures in the Ukrainian setting will be crucial for assessing the true severity of mental health issues and, consequently, for providing children who require assistance with the necessary resources and targeted mental health interventions during and after the war.

Consequently, the objective of the current study was to develop caregiver-reported and age-appropriate versions of the IAQ and IDQ, named as the IAQ-Caregiver Version (IAQ-CG) and the IDQ-Caregiver Version (IDQ-CG). Moreover, this study sought to develop Ukrainian versions of these measures. Furthermore, the current study sought to test the (1) symptom structure of the Ukrainian IAQ-CG and IDQ-CG, (2) reliability of scores from the Ukrainian IAQ-CG and IDQ-CG, (3) examine convergent-divergent validity patterns of the Ukrainian IAQ-CG and IDQ-CG through associations with external measures of internalizing, attention, and externalizing problems, as well as total trauma exposure, and (4) examine differences in IAQ-CG and IDQ-CG average scores in relation to a range of demographic and war-related correlates.

## Methods

### Participants and procedures

Data for the present study was derived from the “*The Mental Health of Parents and Children in Ukraine Study: 2023 Follow-up”* study, that monitors the effects of Russia’s war on Ukraine on the everyday lives and mental health of Ukrainian parents and their children. Participants were recruited by the survey company *TGM Research* between September 7th and September 18th, 2023, using opportunistic sampling techniques because of the ongoing conflict and widespread mass displacement in Ukraine. However, every effort was made to ensure that the sample was as representative of the adult Ukrainian population as was possible. Inclusion criteria for the study included being aged 18 years or older, currently living in Ukraine, having at least one child under the age of 18 years, and being able to complete the survey in Ukrainian.

Data from participants who passed all attention checks was analysed. Of the total sample of 2,050 people, 31.2% (*n* = 639) reported being the parent of a child aged 7 to 17 years, and thus these adults comprised the final sample. The average age of children was 11.67 years (SD = 3.13) and the ratio of males (50.5%; *n* = 323) to females (49.5%; *n* = 316) was relatively equal. Most parents (87.6%; *n* = 560) reported that their child lived with them, while a small proportion reported that their child was living with another parent outside of Ukraine (6.6%; *n* = 42) or elsewhere in Ukraine (5.8%; *n* = 37).

### Measures

The IDQ-CG and IAQ-CG are based on ICD-11 criteria for depression and the items directly map onto the respective criteria. The adult measure of IDQ [9] was used as a basis and child friendly language was used covering the respective developmentally appropriate criteria by three experts in the field of child and adolescent psychiatry/psychotherapy. The first draft was presented to children using the think aloud protocol, whereby children were advised to say whatever comes into their mind as they complete the task. The task was performed with 5 children with the German translated versions of the IAQ and IDQ within a German mental health clinic. After refining the wordings and ensuring to cover all important aspects while using child friendly language the final versions were created. These items were then translated into Ukrainian by one of the study authors that is fluent in Ukrainian and English (DM).

IDQ-CG: The caregiver version of the IDQ-CG was used in this study. The IDQ-CG is a 9-item measure to assess children’s depressive symptoms from a caregiver perspective. Caregivers rated the presence of symptoms in the last two weeks using a four-point Likert scale (0 = Never, 1 = Sometimes, 2 = Often and 3 = Almost Always). The IDQ-CG can be used to calculate a dimensional total score of the nine items (range = 0–27) or following a categorical approach scoring the diagnostic algorithm of the ICD-11 with a symptom present with at least a frequency rating of 2 = Often. In the present study, internal reliability was good/excellent for the total scale (α = 0.88).

IAQ-CG: The caregiver version of the IAQ-CG was used in this study. The IAQ-CG is a 8-item measure to assess children’s anxiety symptoms from a caregiver perspective. Caregivers rated the presence of symptoms in the last two weeks using a four-point Likert scale (0 = Never, 1 = Sometimes, 2 = Often and 3 = Almost Always). The IAQ-CG can be used to calculate a dimensional total score of the eight items (range = 0–24) or following a categorical approach scoring the diagnostic algorithm of the ICD-11 with a symptom present with at least a frequency rating of 2 = Often. In the present study, internal reliability was good/excellent for the total scale (α = 0.89).

Psychosocial functioning: The Paediatric Symptom Checklist (PSC-17) [12] is a 17-item measure designed to identify cognitive, emotional, and behavioural problems. Both caregiver-report and self-report versions of the PSC-17 are available; the former was utilised in the present study. To account for potential changes in emotional or behavioural issues during the past month, the PSC-17 response structure was slightly modified for this study. Caregivers rated the presence of symptoms using a three-point Likert scale (0 = Less Often, 1 = The Same, and 2 = More Often). The PSC-17 can be used to calculate total scores on the attention subscale (range = 0–10), internalizing subscale (range = 0–10), and externalizing subscale (range = 0–14), in addition to a total scale score (range = 0–34). In the present study, internal reliability was excellent for the total scale (α = 0.87), internalizing subscale (α = 0.81), and externalizing scale (α = 0.78), and was adequate for the attention scale (α = 0.73).

Trauma exposure: The Child and Adolescent Trauma Screen 2 (CATS-2) [13] is a 15-item measure designed to measure exposure to traumatic events during childhood. The CATS includes item relating to interpersonal (e.g., ‘seeing someone in the family threatened, hit or hurt badly’) and non-interpersonal traumas (e.g., ‘Threatened, hit, or hurt badly within the family’). Items are scored dichotomously as yes (1) or no (0) responses. For the purposes of the current study, items were summed to determine total amount of trauma exposure.

#### Child-related variables

Child-related variables were reported by parents and included gender (0 = male, 1 = female), age (in years), delayed milestone development (such as delay in speech development or walking without support) (0 = no, 1 = yes), child with prior psychological or pharmacological support for emotional or behavioural problems (0 = no, 1 = yes), bereavement during lifetime (0 = no, 1 = yes).

#### Parent-related variables

Parent-related variables were reported by parents and included forced to move to another part of Ukraine (0 = no, 1 = yes) and serious disruption to life since Russian invasion (0 = no, 1 = yes).

### Analytic procedures

First, item response and summary statistics for the IDQ-CG and IAQ-CG items were calculated. This included item-to-total correlations where values ≥ 0.30 indicated that the scale items were adequately related to all other items in the scales [14].Second, the unidimensionality of the IDQ-CG and IAQ-CG were tested using confirmatory factor analysis (CFA) based on prior studies providing support for a unidimensional structure of the IAQ and IDQ. The IDQ-CG and IAQ-CG allow the summing of items to indicate the severity of symptoms - the “ordinal scoring” method - as well as to evaluate “caseness” or the proportion of participants who meet the diagnostic requirements - the “binary scoring” method. Thus, four one-factor models were tested: for **Model 1** all ordinal IDQ-CG items loaded onto a single ‘depression’ latent variable, for **Model 2** all binary IDQ-CA items loaded onto a single ‘depression’ latent variable, for **Model 3** all ordinal IAQ-CG items loaded onto a single ‘anxiety’ latent variable, and for **Model 4** all binary IAQ-CG items loaded onto a single ‘anxiety’ latent variable. These models were tested using Mplus Version 8.9 [15] with the robust weighted least squares estimator (WLSMV). Model fit was assessed according to standard recommendations [16] where ‘acceptable’ model fit was indicated by a non-significant chi-square value; Comparative Fit Index (CFI) [17] and Tucker-Lewis Index (TLI) [18] values ≥ 0.90; and Root Mean Square Error of Approximation (RMSEA) [19] and Standardized Root Mean Square Residual (SRMR) [20] values ≤ 0.08.

Following identification of the best-fitting CFA model, composite reliability estimates were calculated which provide a more accurate estimation of internal reliability than Cronbach’s alpha [21]. CR values range from 0 to 1, with scores closer to 1 indicating higher internal reliability [21]. For the fourth step, bivariate correlations were calculated between the latent variables derived from the CFA analyses (based on the ordinal items) and age, the PSC-17 subscales (i.e., internalizing, externalizing, and attention score), and total trauma scores. Next, a series of independent samples t-tests were conducted to compare means for the IDQ-CG and IAQ-CG total scores across the categorical variables (i.e., gender, delayed milestone development, child with prior psychological or pharmacological support for emotional or behavioural problems, parent having to relocate to another part of Ukraine, parental distress because of the Russian invasion). To account for multiple comparisons, the alpha level was adjusted to 0.01 (0.05/5). Finally, prevalence rates of probable ICD-11 DD and ICD-11 GAD were estimated.

## Results

### Sample descriptives

Item response and summary statistics for the IDQ-CG and IAQ-CG are presented in Table 1. The observed range of scores on the IDQ-CG was 0 to 20, with a mean score of 3.72 (*SD* = 3.54, Median = 0.00). The observed range of scores on the IAQ-CG was 0 to 22, with a mean score of 4.76 (*SD* = 3.80, Median = 4.00). The distribution was positively skewed for both the IDQ-CG scores (*S* = 1.19, *SD* = 0.10) and IAQ-CG scores (*S* = 0.74, *SD* = 0.10). The most endorsed IAQ-CG items were item 2 (‘My child worries a lot about different things’) and item 6 (‘My child has problems paying attention’). The most endorsed IDQ-CG items were item 3 (‘My child had problems paying attention’) and item 2 (‘My child was bored or didn’t have fun anymore’).

**Table 1:** Item response and summary statistics for the IDQ-CG and IAQ-CG

	Scale value						
	0 Never	1 Sometimes	2 Often	3 Almost always	% Endorsed	Mean (SD)	Item-to-total Correlation
***IAQ-CG items***							
1. My child felt nervous or anxious.	38.2%	53.8%	7.0%	0.9%	8.0%	0.71 (0.64)	0.81
2. My child worried a lot about different things.	35.8%	53.7%	9.9%	0.6%	10.5%	0.75 (0.65)	0.78
3. My child felt stressed or upset.	41.3%	50.7%	7.4%	0.6%	8.0%	0.67 (0.64)	0.77
4. My child felt strong feelings in her/his body (heart beating, difficulty breathing, upset stomach, sweating or dry mouth).	70.4%	26.6%	2.3%	0.6%	3.0%	0.33 (0.55)	0.71
5. My child felt restless or jumpy.	62.8%	32.2%	4.7%	0.3%	5.0%	0.43 (0.60)	0.76
6. My child had problems paying attention.	39.4%	50.1%	8.6%	1.9%	10.5%	0.73 (0.70)	0.72
7. My child felt easily annoyed, grumpy or mad.	48.2%	43.7%	7.2%	0.9%	8.1%	0.61 (0.66)	0.75
8. My child had trouble falling or staying asleep.	55.2%	37.4%	6.4%	0.9%	7.4%	0.53 (0.66)	0.68
***IDQ-CG items***							
1. My child felt sad or unhappy.	47.9%	48.0%	3.4%	0.6%	4.1%	0.57 (0.59)	0.73
2. My child was bored or didn’t have fun anymore.	42.9%	51.5%	5.2%	0.5%	5.6%	0.63 (0.60)	0.75
3. My child had problems paying attention.	39.9%	49.8%	8.9%	1.4%	10.3%	0.72 (0.68)	0.72
4. My child felt worthless or blamed himself/herself.	77.6%	19.7%	2.3%	0.3%	2.7%	0.25 (0.51)	0.73
5. My child felt hopeless.	80.4%	17.7%	1.7%	0.2%	1.9%	0.22 (0.46)	0.71
6. My child thought about death or killing himself/herself.	92.2%	6.7%	1.1%	0.0%	1.1%	0.09 (0.32)	0.51
7. My child had problems with his/her appetite or sleep.	56.0%	38.8%	4.1%	1.1%	5.2%	0.50 (0.63)	0.70
8. My child was moving slower or felt more restless.	71.4%	25.7%	2.8%	0.2%	3.0%	0.32 (0.53)	0.78
9. My child had no energy or felt tired (just sat around or did nothing)	62.8%	32.7%	4.2%	0.3%	4.5%	0.42 (0.59)	0.76

Please note: All item-to-total correlations are significant at *p* < .001

Item-to-total correlations for the IDQ-CG items ranged from 0.51 for item 6 (‘My child had thoughts about death or killing himself/herself’) to 0.78 for item 8 (‘My child was moving slower or felt more restless’), and for the IAQ-CG items values ranged from 0.68 for item 8 (‘My child had trouble falling or staying asleep) to .81 for item 1 (‘My child felt nervous or anxious’).

As shown in Table 4, there was no statistically significant difference in mean IDQ-CG scores for males (*M* = 3.58, SD = 3.48) and females (*M =* 3.86, SD = 3.59), t (637) = -1.01, *p* = .157. There was also no statistically significant difference in mean IAQ-CG scores for males (*M* = 4.78, SD = 3.93) and females (*M =* 4.74, SD = 3.66), t (637) = 0.14, *p* = .443.

### CFA results and reliability

Table 2 provides the CFA fit statistics for IDQ-CG and IAQ-CG models using both the ordinal items and binary items. Findings illustrated that the one-factor model using ordinal items provided a reasonable fit to the data for both the IDQ-CG and IAQ-CG. Although the chi-square statistic was significant for both models, this should not be taken as evidence to reject the models as the chi square statistic is positively associated with sample size (Tanaka, 1987). Thus, the chi-square statistic was divided by degrees of freedom and this resulted in chi-square values of 7.36 and 7.73 for the IDQ-CG and IAQ-CG, respectively. The RMSEA for both models was higher than the conventional cut-off for ‘close fit’ of below 0.08. This may be attributable to these models having few indicators with high factor loadings (Shi et al., 2019), but model misspecification was also assessed by inspecting the modification indices (MI). For the IAQ-CG the fit improved with the addition of the parameter with the largest MI; a correlated error was added between items 4 and 5 and the fit statistics improved (χ^2^ = 95.09, df = 19, p = < 0.001; TLI = 0.985; CFI = 0.990; RMSEA = 0.079 (95% CI = 0.06, 0.10); SRMR = 0.03). For the IDQ-CG, the fit improved with the addition of the parameter with the largest MI; a correlated error was added between items 4 and 5 and the fit statistics improved (χ^2^ = 127.015, df = 26, *p* < .001; TLI = 0.981; CFI = 0.986; RMSEA = 0.078 (95% CI = 0.065, 0.092); SRMR = 0.047). For the IDQ-CG and IAQ-CG, all factor loadings were strong, positive and statistically significant. Factor loadings ranged from 0.73 to 0.90 and from 0.69 to 0.90 for the IDQ-CG and IAQ-CG, respectively.

**Table 2:** Fit Statistics for CFA models

Scale	Item format	χ^2^ (df), *p*	TLI	CFI	RMSEA (90% C.I.)	SRMR
Depression (*n* = 639)	Continuous	198.852 (27), *p* < .001	0.969	0.977	0.100 (0.087, 0.113)	0.053
	Binary	42.906 (27), *p* = .027	0.983	0.987	0.030 (0.011, 0.047)	0.081
Generalized Anxiety (*n* = 639)	Continuous	154.618 (20), *p* < .001	0.975	0.982	0.103 (0.088, 0.118)	0.034
	Binary	40.574 (20), *p* = .004	0.988	0.991	0.040 (0.022, 0.058)	0.061

*Note* χ^2^ = chi-square test, TLI = Tucker Lewis Index, CFI = Comparative Fit Index, RMSEA = Root Mean Square Error of Approximation, Standardized Root Mean Square Residual

Findings also illustrated that the one-factor model using binary scores provided an acceptable fit to the data for both the IDQ-CG and IAQ-CG. For the IDQ-CG and IAQ-CG, all factor loadings were strong, positive, and statistically significant. Factor loadings ranged from 0.74 to 0.95 and from 0.73 to 0.94 for the IDQ-CG and IAQ-CG, respectively.

Composite reliability estimates indicated that the internal reliability was excellent for the IDQ-CG using the ordinal items (0.95) and binary items (0.96). Similarly, composite reliability estimates indicated that the internal reliability was excellent for the IAQ-CG using ordinal items (0.94) and binary items (0.96).

### Associations with age and external variables

As shown in Table 3, age was not associated with any of the latent variables. However, there were moderate-to-strong, positive, and statistically significant associations between attention, internalizing, externalizing scores and total trauma scores and all the latent variables (using both ordinal items and binary items).

**Table 3:** Standardized bivariate correlations for the latent factors and mental health outcomes

IAQ-CG (*n* = 639)	Age	Attention	Internalizing	Externalizing	Total trauma
Ordinal item scores	0.049	0.556***	0.750***	0.578***	0.464***
**IDQ-CG** (***n* = 639**)	Age	Attention	Internalizing	Externalizing	Total trauma
Ordinal item scores	0.042	0.494***	0.759***	0.550***	0.391***

*Note* ***p < .001

#### Group differences in average IDQ-CG and IAQ-CG scores

As shown in Table 4, there was no association between gender and total scores on the IAQ-CG and IDQ-CG. There was a significant association between prior psychological or pharmacological support for emotional or behavioural problems, delayed milestone development, parents being forced to move to another part of Ukraine, parents reporting serious life disruption due to the Russian invasion, and having experienced a bereavement and total scores on the IAQ-CG and IDQ-CG.

**Table 4:** Group differences in average IDQ-CG and IAQ-CG scale scores

			IDQ-CG					IAQ-CG				
Variables	Group	*N*	M	SD	DF	t	d	M	SD	DF	t	d
Gender	Male	323	3.58	3.58	637	-1.01	-	4.78	3.93	637	0.14	-
	Female	316	3.86	3.86				4.74	3.66			
Delayed milestone development	Yes	89	5.70	4.37	105.64	**-4.85**	0.66	6.79	4.49	106.81	-4.72	0.63
	No	550	3.40	3.30				4.43	3.57			
Prior psychological/pharmacological support	Yes	55	7.63	4.47	59.65	**-7.53**	1.46	9.40	3.99	62.47	**-9.96**	1.66
	No	484	2.98	3.00				3.84	3.26			
Parent forced to move to another part of Ukraine?	Yes	108	4.69	3.99	140.53	**-2.83**	0.33	5.76	4.06	637	**-3.02**	0.31
	No	531	3.52	3.41				4.56	3.72			
Parent’s life been seriously disrupted in any way since the Russian invasion	Yes	283	4.11	3.93	534.93	**-2.45**	0.21	5.20	4.09	558.44	**-2.57**	5.20
	No	356	3.40	3.16				4.41	3.52			
Bereavement	Yes	277	5.16	3.96	637	**-2.34**	0.19	4.12	3.66	637	**-2.52**	0.20
	No	362	4.45	3.65				3.41	3.41			

*Note* t-values in bold are significant at *p* < .001

### Prevalence estimates

In total, 1.6% (95% CI = 0.6%, 2.5%) of young people met diagnostic requirements for ICD-11 DD and 5.8% (95% CI = 4.0%, 7.6%) for ICD-11 GAD, based on parental reports. Of those who satisfied the requirements for either disorder, 1.3% (*n* = 8) met diagnostic requirements for both ICD-11 DD and ICD-11 GAD. There was no statistically significant association between child sex and meeting diagnostic requirements for either ICD-11 DD (*χ*^*2*^ (1) = 0.363, *p* = .547) or ICD-11 GAD (*χ*^*2*^ (1) = 0.606, *p* = .436).

## Discussion

The primary aim of this study was to test the psychometric properties of Ukrainian caregiver-reported versions of the IDQ and IAQ for children and adolescents, referred to as the IDQ-CG and IAQ-CG, respectively. The IDQ-CG and IAQ-CG were developed based on the adult measures of the IDQ and IAQ [10], and child friendly language was used covering the respective developmentally appropriate criteria by three experts in the field of child and adolescent psychiatry/psychotherapy and based on presenting these measures to a sample of children. Findings provide initial support for the reliability and validity of the IAQ-CG and IDQ-CG scores and indicate that these measures can be used to identify Ukrainian children and adolescents with clinically relevant depression and anxiety symptoms, respectively.

Consistent with prior research supporting a unidimensional representation of the IAQ and IDQ [10], CFA results that the one-factor model provided a good fit to the data for both measures using the ordinal items and binary items. All IDQ-CG and IAQ-CG items loaded strongly and significantly onto their respective latent variables, showing that they are excellent indicators of their respective latent variables. This is particularly important given that the items contained within the original IDQ and IAQ were amended to reflect how symptoms are anticipated to manifest in children between the ages of 7 and 17. Aligning with prior research (e.g., 10; 12), the internal reliability of the IDQ-CG and IAQ-CG was excellent. Moreover, the patterns of association between IAQ-CG and IDQ-CG latent variables and their correlates provided support for the convergent validity of these measures. Specifically, there were strong associations with internalising scores, and moderate associations with attention, externalising, and total trauma scores. Overall, these findings provide support for the validity and reliability of the IDQ-CG and IAQ-CG.

The current study also sought to explore the association between a range of correlates and IAQ-CG and IDQ-CG scores. Findings demonstrated a significant association between prior psychological or pharmacological support for emotional or behavioural problems and total scores on both measures. This is anticipated considering that the psychological effects of being exposed to traumatic events, such as war, are typically more severe for people with pre-existing mental health problems [22]. Furthermore, the complexities of the war have led to a significant depletion in the availability of psychiatric medications, exacerbating symptoms in young people with pre-existing mood and anxiety disorders [3]. In line with prior studies indicating heightened anxiety and depression symptoms in young people experiencing delays in achieving their developmental milestones [23], this study revealed a notable correlation between delayed milestone attainment and levels of anxiety and depression symptoms. Children respond differently to the stress of violent experiences based on their level of social-emotional and cognitive development [24], likely explaining this association. Finally, consistent with prior research demonstrating how stressful life events and daily hardships can result in greater internalizing symptomology in young people [e.g., [25]], our findings show that being forced to move to another part of Ukraine, serious life disruption due to the Russian invasion, and experiencing a bereavement were significantly associated with total scores on the IDQ-CG and IAQ-CG. Overall, the identification of risk factors associated with increased levels of anxiety and depression in Ukrainian young people will facilitate the identification of those young people in greatest need of mental health support.

The final aim of the current study was to estimate the proportion of children that meet diagnostic requirements of ICD-11 GAD and ICD-11 DD based on parental-reports. Findings demonstrated that 1.6% of the sample met diagnostic requirements for probable ICD-11 DD and 5.8% met diagnostic requirements for probable ICD-11 GAD. These rates are notably lower than adult studies using the self-reported IAQ and IDQ where rates of ICD-11 GAD ranged from 7.1 to 28.5% and rates of ICD-11 DD 7.4–26.6% [9–11]. Prior research has shown how caregivers often underestimate internalising symptoms in their children [26], and thus, it is possible that the caregiver-reported nature of the measures in the present study may have led to an underestimation of the true prevalence of these disorders in young people. Nevertheless, findings indicate that a small proportion of young Ukrainians fulfil the diagnostic requirements for ICD-11 GAD and ICD-11 DD.

There are several limitations which should be considered when interpreting findings from the present study. First, due to the ongoing conflict and the significant mass displacement in Ukraine, the sample was not selected using random probability sampling; as a result, findings might not be generalizable. Nevertheless, efforts were made to ensure that the sample was as representative as it could be. Second, it was not possible to collect data from regions under Russian occupation. Prior research has shown elevated rates of depression and anxiety in young people living in more heavily affected regions in Ukraine [5], and thus, the prevalence estimates in the current study may not provide an accurate reflection of the extent of these issues in all Ukrainian young people. Third, inferences regarding causality or temporal ordering cannot be drawn from this study due to it being a cross-sectional study. Finally, the IAQ-CG and IDQ-CG were developed as screening instruments and thus, should be used in conjunction with a thorough clinical assessment.

To conclude, this study provides initial support for the reliability and validity of the the caregiver-report version of the International Depression Questionnaire (IDQ-CG) and International Anxiety Questionnaire (IAQ-CG). The availability of empirically validated Ukrainian translated measures of depression and anxiety which align with current diagnostic guidelines will be instrumental with respect to mental health screening and evaluating the need for mental health services in Ukraine [3]. These measures will also be extremely useful for mental health providers in neighboring countries hosting young Ukrainian refugees. From a research perspective, having these measures available will promote research on the mental health of Ukrainian refugees, an issue that has not received sufficient attention within the literature [27]. Beyond the war context, the caregiver-reported nature of the IDQ-CG and IAQ-CG will be hugely beneficial when a child’s reading abilities are inadequate, or when clinicians require additional information to supplement their evaluations of child and adolescent self-report information. Indeed, caregivers possess the most in-depth knowledge of their child’s development and can report on their child in a manner that is sensitive to contextual, historical, and developmental factors [28].

## Data Availability

Neither the data nor the materials have been made available on a permanent third-party archive.
